# Spatiotemporal pattern of leprosy in southwest China from 2010 to 2020: an ecological study

**DOI:** 10.1186/s12889-024-17859-6

**Published:** 2024-02-14

**Authors:** Mengyan Zhang, Longchong Qiao, Peiwen Sun, Haiqin Jiang, Ying Shi, Wenyue Zhang, Youming Mei, Meiwen Yu, Hongsheng Wang

**Affiliations:** 1 Department of Epidemiology and Biostatistics, School of Public Health, Nanjing Medical University, Nanjing, Jiangsu China; 2 Institute of Dermatology, Chinese Academy of Medical Sciences & Peking Union Medical College, Nanjing, Jiangsu China; 3 National Centre for Leprosy Control, China CDC, Nanjing, Jiangsu China; 4 Jiangsu Key Laboratory of Molecular Biology for Skin Diseases and STIs, Nanjing, Jiangsu China

**Keywords:** Leprosy, Epidemiology, Temporal trends, Cluster, China

## Abstract

**Background:**

Despite many efforts to control leprosy worldwide, it is still a significant public health problem in low- and middle-income regions. It has been endemic in China for thousands of years, and southwest China has the highest leprosy burden in the country.

**Methods:**

This observational study was conducted with all newly detected leprosy cases in southwest China from 2010 to 2020. Data were extracted from the Leprosy Management Information System (LEPMIS) database in China. The Joinpoint model was used to determine the time trends in the study area. Spatial autocorrelation statistics was performed to understand spatial distribution of leprosy cases. Spatial scan statistics was applied to identify significant clusters with high rate.

**Results:**

A total of 4801 newly detected leprosy cases were reported in southwest China over 11 years. The temporal trends declined stably. The new case detection rate (NCDR) dropped from 4.38/1,000,000 population in 2010 to 1.25/1,000,000 population in 2020, with an average decrease of 12.24% (95% CI: −14.0 to − 10.5; *P* < 0.001). Results of global spatial autocorrelation showed that leprosy cases presented clustering distribution in the study area. Most likely clusters were identified during the study period and were frequently located at Yunnan or the border areas between Yunnan and Guizhou Provinces. Secondary clusters were always located in the western counties, the border areas between Yunnan and Sichuan Provinces.

**Conclusions:**

Geographic regions characterized by clusters with high rates were considered as leprosy high-risk areas. The findings of this study could be used to design leprosy control measures and provide indications to strengthen the surveillance of high-risk areas. These areas should be prioritized in the allocation of resources.

## Background

 Leprosy, or Hansen’s disease [1], caused by *Mycobacterium leprae(M. leprae)* [2], is one of twenty Neglected Tropical Diseases (NTDs) according to the classification proposed by the World Health Organization (WHO). Its occurrence is often thought to be related to poor socioeconomic conditions [3]. Bacteria are disseminated by droplets from the nose and mouth during close or frequent contact with untreated cases. Once a person is infected, the skin and peripheral nerves are mainly affected [4]. If not treated at the early stage, then it will lead to progressive disability and permanent disfigurement of the affected individual, resulting in social stigma [4, 5].

After WHO launched multidrug therapy (MDT: composed of rifampicin, clofazimine and dapsone) in the 1980s, leprosy prevalence declined dramatically across the globe [6]. “The leprosy prevalence rate < 1 case/10,000 population” was defined as leprosy elimination [7]. Although some regions have achieved this goal, there are still some regions and countries with relatively high burden of leprosy. The Weekly epidemiological record published by WHO reported that 140,594 new cases were recorded globally in 2021, most coming from the less economically developed regions; 66.5% was contributed by Southeast Asia, followed by 15.1% from Africa. Brazil, India, and Indonesia continued to the top three countries struggling with leprosy, reporting more than 10,000 new cases [8].  

Historically, China had also made great efforts to eradicate leprosy. In 1990, the National Leprosy Recordings and Reporting System was established by the Ministry of Health, which was used to collect clinical and epidemiological records of all leprosy cases [9]. In 2011, the National Leprosy Elimination Program (NLEP) (2011–2020) was proposed to reduce the effects of leprosy as soon as possible [10]. The prevalence and epidemic range of leprosy were reduced, and the harm to patients was also decreased. Despite many efforts, some areas in China still struggle with the leprosy endemic, especially southwest China [11]. More than 50% leprosy cases in China occurred in this region, although the population of this area is less than 10% of the whole nation. Hence, identifying high-risk areas of epidemiological significance is essential, which is conducive to designing public health measures and guiding the interventions.

In recent years, a series of spatial and temporal methods has been extensively applied in understanding the distribution [12] and transmission [13] of infectious diseases. Several studies [10, 14] described the epidemiological features of leprosy only in a certain province of China. No studies have observed the spatial or spatiotemporal clusters across 11 years in southwest China.

The aims of this study are as follows. First, it aims to understand the spatial and temporal characteristics of leprosy in southwest China from 2010 to 2020. Second, it aims to identify the high-risk areas for leprosy transmission. The findings of this study could guide the allocation of resources in southwest China and provide evidence to design public health polies for improved leprosy control.

## Methods

### Study area

This study was conducted in southwest China, which is located between the latitudes of 21°8′–34°19′N and longitudes of 97°31′–109°35′E. Southwest China consists of 400 counties (Yunnan: 129 counties; Guizhou: 88counties; Sichuan: 183 counties). Its territory spans 1,056,267 km^2^, and its total population was 130,697,188 in the 2020 national population census. In the last decade, more than 50% of leprosy cases in China occurred in this region. The economy in this region is also comparatively backward.

### Data sources

The data of all confirmed new leprosy cases in the study area from January 1, 2010, to December 31, 2020, were collected from the Leprosy Management Information System (LEPMIS) database in China. Newly detected leprosy cases were clinically diagnosed by medical staff specializing in leprosy and certified by the provincial CDC in each region. The diagnostic criteria for leprosy have remained almost unchanged for the last 20 years. The criteria are at least one of the following cardinal signs: (1) definite loss of sensation in a pale (hypopigmented) or reddish skin patch; (2) thickened or enlarged peripheral nerve, with loss of sensation and/or weakness of the muscles supplied by that nerve; (3) microscopic detection of bacilli in a slit-skin smear [15]. Each leprosy case record included epidemiological and clinical information, such as case ID, age, sex, ethnicity, education, classification, date of birth, date of onset, date of diagnosis, and precise address to the county. The population data, available to calculate the new case detection rate (NCDR), were obtained from China Statistical Yearbook. The following equation was used to calculate the NCDR:

$$\text{NCDR}=\frac{\text{Newly detected leprosy cases}}{\mathrm{population\ in\ the\ area\ and\ period}}$$

### Statistical data analysis

#### Time trend analysis using Joinpoint

To determine the temporal trends during study period, we performed joinpoint regression by Joinpoint software, version4.9.1.0, (National Cancer Institute, United States). According to Kim et al. [16], who proposed of this method, the Monte Carlo permutation test was performed to define the best-fitting points significantly (*P* < 0.05), which are called “joinpoints”. The Z test was used to estimate the annual percent change (APC) of the slope for each segment between the joinpoints and calculate the overall average annual percent change (AAPC) with 95% confidence interval (CI). However, when the joinpoints are zero, AAPC is identical to APC. The trends are described by using the terms “increasing” and “decreasing” to indicate the slope at significance. Conversely, “stable” means the slope at no significance.

#### Global spatial autocorrelation analysis using GeoDa

The spatial autocorrelation statistic was evaluated by using the global Moran’s index (Moran’s *I*), assessing the general spatial correlation throughout the study region, which is between − 1 and 1. Its value is closer to 1, indicating that the distribution of leprosy is more spatially clustered. We created a spatial weight matrix by using Queen Contiguity method to calculate the global Moran’s *I*. We then used the empirical Bayesian model was used to correct the random fluctuation caused by a small population or low numbers of newly leprosy cases [17]. The global Moran’s *I* were calculated by using the following formula:

$$\mathrm{Global\ Moran}'\mathrm{s\ I}\;=\;\frac{\mathrm{n}}{{\mathrm{S}}_0}\;\times\;\frac{\sum_{\mathrm{ i}=1}^{\mathrm{n}}\;\sum_{\mathrm{j}=1}^{\mathrm{n}}\;{\mathrm{w}}_{\mathrm{ij}}\;\left({\mathrm{y}}_{\mathrm{i}}-\overline{\mathrm{y}}\right)\;\left({\mathrm{y}}_{\mathrm{j}}-\overline{\mathrm{y}}\right)}{\sum_{\mathrm{i}=1}^{\mathrm{n}}\;\left({\mathrm{y}}_{\mathrm{i}}-\overline{\mathrm{y}}\right)^2}$$

Where *S*_0_ is the aggregation of all spatial weights, W_i j_ is the spatial weight between regions i and j; n is the total number of spatial elements; ${\text{y}}_{\text{i}}$ and ${\text{y}}_{\text{j}}$ represent the attribute values for region i or j; and $\stackrel{\text{-}}{\mathrm{y}}$ is the average for attribute values of all spatial elements.

#### Purely- spatial and space-time analysis using SaTScan

Leprosy clusters with high rates were detected by conducting purely spatial and space-time scan statistics by using SaTScan software, version 10.0 (https://www.satscan.org/) based on the maximum likelihood method and the Poisson discrete model. The model evaluates the relative risk (RR) for the identified cluster areas. Monte Carlo simulations (using 999 permutations) were performed to define the *P* value, and clusters with *P* < 0.05 were considered significant.

The principle of purely spatial scan statistics is to set up a circular window on the map; this window varies continuously in size and position. The radius of the window is set as 15% of the total population at risk according previous research [18, 19]. Similar to purely spatial statistics, space-time scan statistics is defined by a cylindrical window on the map. Then, the cylindrical window moves simultaneously in space and time to identify clusters. It is worth noting that the most likely clusters and secondary clusters are independent of each other. Generally, areas characterized by most likely clusters are supposed to have the highest priority for intervention.

## Results

### Description of leprosy data

**Fig. 1:**
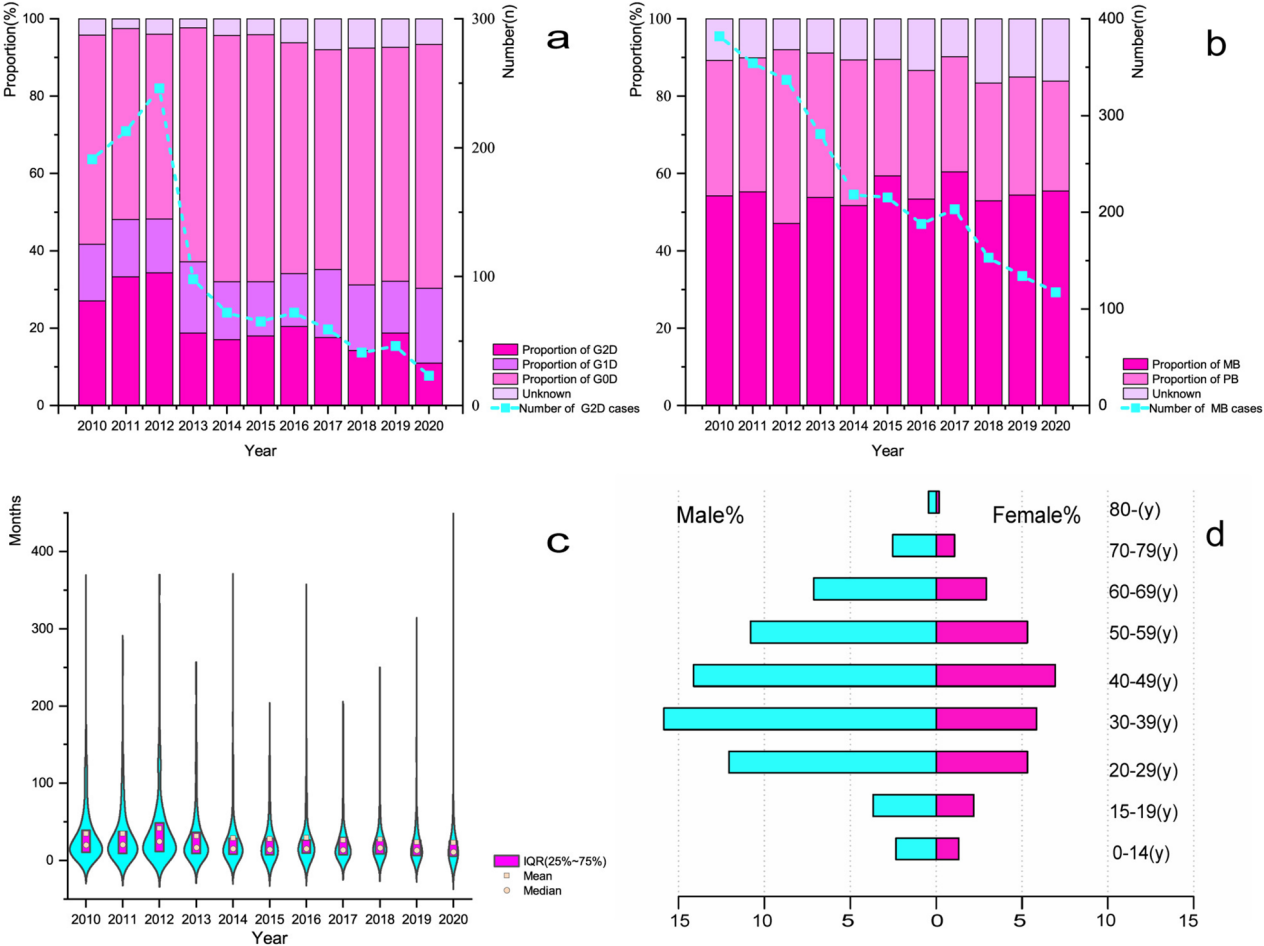
Epidemiological and demographic characteristics of newly detected leprosy cases in southwest China, 2010 to 2020. **a** Epidemiological characteristics of disability. **b** Epidemiological characteristics of clinical type. **c** The delayed diagnosis time of newly detected leprosy cases. **d** Age and gender distribution of newly detected leprosy cases

Over 11 years from 2010 to 2020, 4801 newly detected leprosy cases from 400 counties were recorded in southwest China, with an average case detection rate of 2.64/1,000,000 person-year (Table 1; Fig. 1). With regard to the epidemiological characteristics, 1126 (23.45%) newly detected leprosy patients in southwest China were diagnosed with grade 2 disability(G2D). The number of G2D patients increased from 191 (27.09%) in 2010 to 246 (34.36%) in 2012 and then decreased to 23 (10.90%) in 2020. More than half of the newly detected leprosy cases (53.78%; *n* = 2582) were classified as multibacillary (MB). The annual proportion of MB cases exhibited minimal fluctuations. During the study period, the delay time in diagnosis was almost stable annually, with mean (31.99 ± 45.94) and median (17.10 [8.50–35.13]) months overall. Considering the sociodemographic characteristics, 1,492(31.08%) leprosy cases were females. The cases were predominantly males (68.92%; *n* = 3309). A total of 175 (3.65%) new leprosy patients were under the age of 15. Most new leprosy patients (82.11%; *n* = 3942) aged 15 to 59 years. As shown in Fig. 1d, patients aged 30–49 occupied the largest proportion, especially among males.

**Table 1:** Epidemiological and demographic characteristics of newly detected leprosy cases in southwest China, 2010 to 2020

**Year**	**New cases detected**		**G2D**		**MB**		**Female**		**Children**		**Delayed diagnosis months**		
	n	NCDR	n	%	N	%	n	%	n	%	Mean ± SD	Median(IQR)	
2010	705	4.38	191	27.09	382	54.18	208	29.50	31	4.40	34.86 ± 47.79	19.93(10.33-39.00)	
2011	640	3.98	213	33.28	354	55.31	201	31.41	23	3.59	34.82 ± 50.50	20.35(9.25–37.56)	
2012	716	4.44	246	34.36	337	47.07	206	28.77	30	4.19	41.89 ± 53.82	24.67(11.92–48.73)	
2013	522	3.24	98	18.77	281	53.83	168	32.18	15	2.87	31.72 ± 43.80	16.67(9.08–36.21)	
2014	422	2.62	72	17.06	218	51.66	131	31.04	16	3.79	28.78 ± 42.99	15.27(8.12–28.13)	
2015	362	2.14	65	17.96	215	59.39	106	29.28	17	4.70	27.76 ± 45.37	14.22(7.33–28.64)	
2016	352	2.08	72	20.45	188	53.41	117	33.24	16	4.55	29.79 ± 44.74	15.50(9.67–26.40)	
2017	336	1.99	59	17.56	203	60.42	110	32.74	11	3.27	26.32 ± 34.11	13.72(7.33–29.70)	
2018	289	1.71	41	14.19	153	52.94	116	40.14	8	2.77	27.22 ± 35.65	16.53(8.63–26.33)	
2019	246	1.45	46	18.70	134	54.47	65	26.42	4	1.63	23.97 ± 34.33	13.22(6.23–25.87)	
2020	211	1.25	23	10.90	117	55.45	64	30.33	4	1.90	23.16 ± 43.18	11.37(5.17–24.82)	
Total	4801	2.64^a^	1126	23.45	2582	53.78	1492	31.08	175	3.65	31.99 ± 45.94	17.10(8.50-35.13)	

NCDR: (/1,000,000population)

^a^person-year

### Time trends of leprosy cases

**Fig. 2:**
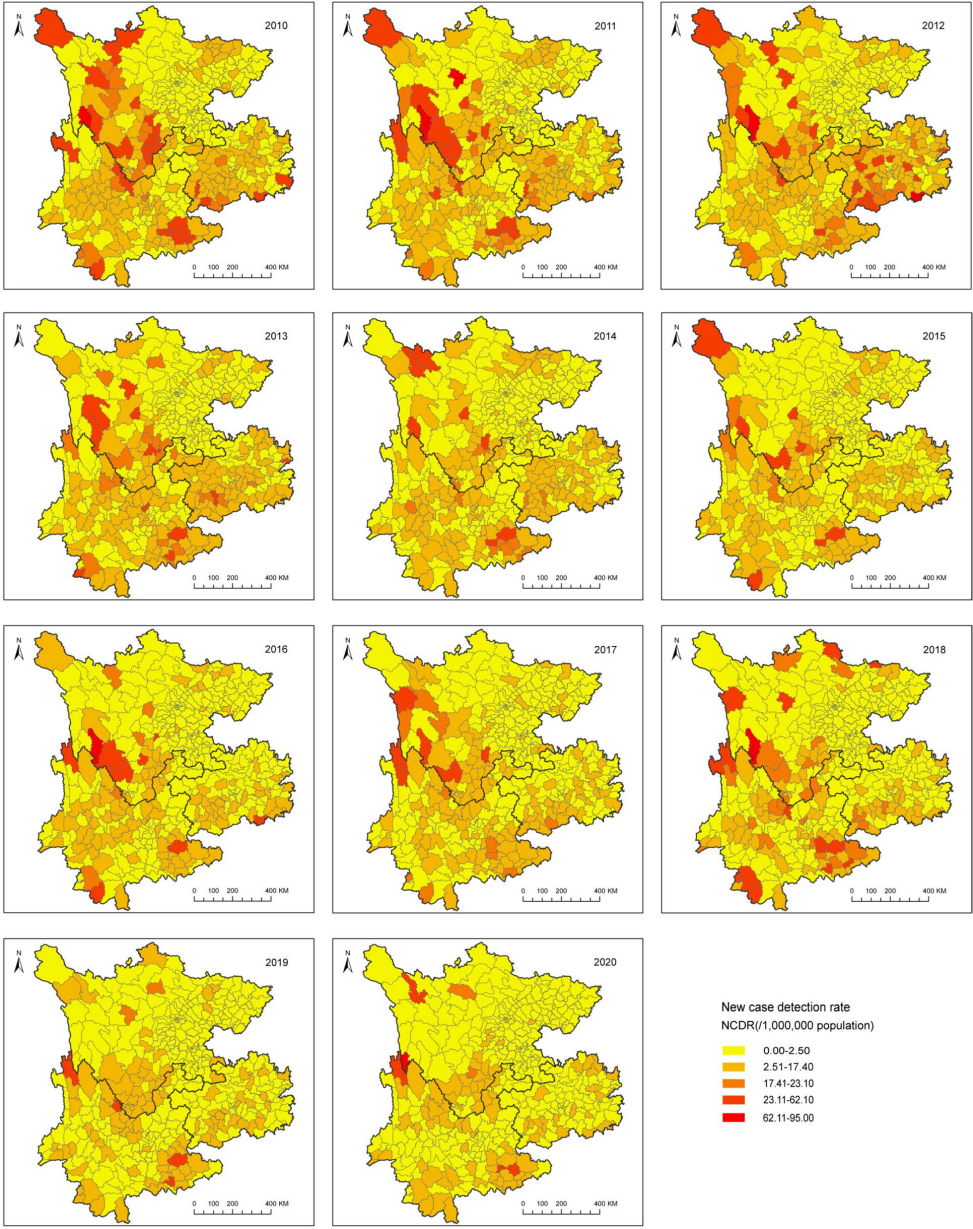
Spatial distribution of NCDR of leprosy in southwest China from 2010 to 2020

The NCDR declined from 4.38 per 1,000,000 population in 2010 to 1.25 per 1,000,000 population in 2020, with an average decline of 12.24% (95% CI: −14.0 to − 10.5) and *P* < 0.001 (Table 2). Nearly half of the newly detected leprosy cases were reported in Yunnan Province (45.84%; *n* = 2201) followed by Guizhou Province (38.43%; *n* = 1845) and Sichuan province (23.23%; *n* = 1115). The three provinces are geographically connected. The annual NCDR in Yunnan was relatively the highest during study time-period, except in 2012, when the NCDR in Guizhou reached the peak. The NCDR in Yunnan ranged from 6.40 in 2010 to 2.54 per 1,000,000 population in 2020, with an average decline of 8.38% (95% CI: − 9.7 to − 7.0) and *P* < 0.001. The NCDR in Guizhou ranged from 5.90 in 2010 to 10.33 per 1,000,000 population in 2012, declining from 10.33 in 2012 to 1.22 per 1,000,000 population in 2020 with an average decline of 17.73% (95% CI: − 22.5 to − 12.6) and *P* < 0.001. NCDR exhibited an average decrease of 12.66% (95% CI; APC: − 15.2 to − 10.0; *P* < 0.001) in Sichuan, from 2.56 in 2010 to 0.54 per 1,000,000 population in 2020. Consequently, the temporal trends of NCDR in the study region was decreasing globally and locally (Fig. 2).

**Table 2:** Temporal trends of newly detected leprosy cases in southwest China

Area/Year	Newly detected leprosy cases (NCDR(/1,000,000population))											Trend			
	2010	2011	2012	2013	2014	2015	2016	2017	2018	2019	2020	APC%	95%CI	*P*-value	Trend
**Yunnan**	294(6.40)	283(6.16)	230(5.00)	241(5.25)	208(4.53)	187(3.99)	170(3.62)	159(3.39)	174(3.71)	136(2.90)	119(2.54)	-8.38	( -9.7, -7.0)	<0.001	Decreasing
**Guizhou**	205(5.90)	208(5.99)	359(10.33)	174(5.01)	123(3.54)	78(2.02)	96(2.49)	81(2.10)	61(1.58)	53(1.38)	47(1.22)	-17.73	(-22.5, -12.6)	<0.001	Decreasing
**Sichuan**	206(2.56)	149(1.85)	127(1.58)	107(1.33)	91(1.13)	97(1.16)	86(1.03)	96(1.15)	54(0.65)	57(0.68)	45(0.54)	-12.66	(-15.2, -10.0)	<0.001	Decreasing
**Total**	705(4.38)	640(3.97)	716(4.44)	522(3.24)	422(2.62)	362(2.14)	352(2.08)	336(1.99)	289(1.71)	246(1.45)	211(1.25)	-12.24	(-14.0, -10.5)	<0.001	Decreasing

### Global spatial autocorrelation of leprosy cases

Based on the annual leprosy raw NCDR, the global spatial autocorrelation results indicated statistical significance (*P* < 0.05) in southwest China across study periods, except in 2020 (Table 3). However, when the empirical Bayesian model was used to lessen the random fluctuation caused by small populations or low numbers of new leprosy cases in some areas, the Moran’s *I* value of the NCDR in 2020 was statistically significant (*P* < 0.001). The finding demonstrated that the distribution of leprosy cases in the study region was very likely clustered at the county-level from 2010 to 2020.

**Table 3:** The global spatial autocorrelation of leprosy cases in southwest China, 2010–2020

Year	Raw NCDR			NCDR smoothed by EB model		
	Moran’s I	Z	P	Moran’s I	Z	P
2010	0.36	9.08	0.001	0.43	10.90	0.001
2011	0.28	8.16	0.001	0.36	9.38	0.001
2012	0.26	6.99	0.001	0.34	8.93	0.001
2013	0.28	7.55	0.001	0.37	9.62	0.001
2014	0.24	6.20	0.001	0.32	8.32	0.001
2015	0.16	4.50	0.003	0.27	7.61	0.001
2016	0.09	3.22	0.012	0.16	4.83	0.001
2017	0.14	3.71	0.005	0.27	7.19	0.001
2018	0.18	4.87	0.003	0.28	7.75	0.001
2019	0.17	4.67	0.002	0.34	9.09	0.001
2020	0.01	0.51	0.167	0.26	6.97	0.001

*NCDR* New case detection rate, *EB model* Empirical Bayesian model

### Spatial clusters of leprosy from 2010 to 2020

**Fig. 3:**
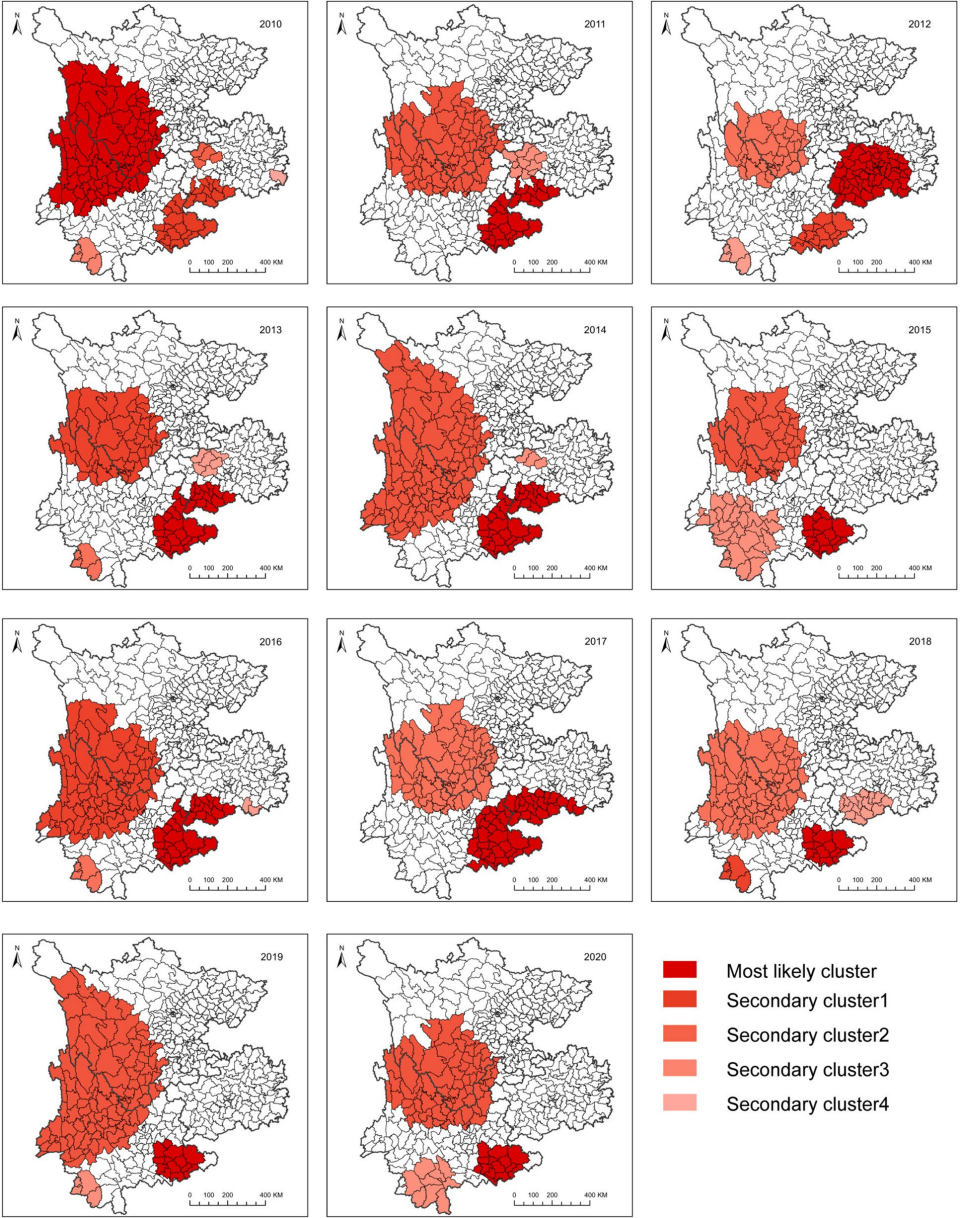
Spatial distribution of leprosy clusters detected by purely spatial scan statistics in southwest China from 2010 to 2020

The purely spatial scan analysis revealed most likely and secondary clusters. Figure 3 shows that the statistically significant clusters vary from 2010 to 2020, but except in 2010, the most likely cluster was mainly concentrated in Yunnan or the border areas between Yunnan and Guizhou Provinces annually. The number of most likely cluster locations was stable among 12 to 80 counties for the 11 years (Table 4). Almost every year, the most likely cluster included Kaiyuan, Luxi, Mengzi, Mile, Guangnan, Malipo, Maguan, Qiubei, Wenshan, Xichou, Yanshan, and Pingbian Miao Autonomous Counties. The secondary clusters were annually located at western region, the border areas between Yunnan and Sichuan Provinces, as indicated by annual results. The number of secondary cluster locations was stable among 36 to 104 counties for the 11 years.

**Table 4:** The number of counties characterized by significant clusters

	Yunnan		Guizhou		Sichuan		Total	
Year	N1	N2	N1	N2	N1	N2	N1	N2
2010	39	20	0	16	41	0	80	36
2011	16	37	10	11	0	36	26	84
2012	0	28	53	0	0	23	53	51
2013	17	4	12	6	0	37	29	47
2014	17	56	12	3	0	45	29	104
2015	14	31	0	0	0	29	14	60
2016	18	56	12	2	0	29	30	87
2017	27	30	21	0	0	35	48	65
2018	12	50	0	15	0	23	12	88
2019	12	58	0	0	0	45	12	103
2020	14	45	0	0	0	36	14	81

N1: The number of counties characterized by most likely clusters

N2: The number of counties characterized by secondary clusters

### Frequency of most likely spatial cluster occurrence from 2010 to 2020

**Fig. 4:**
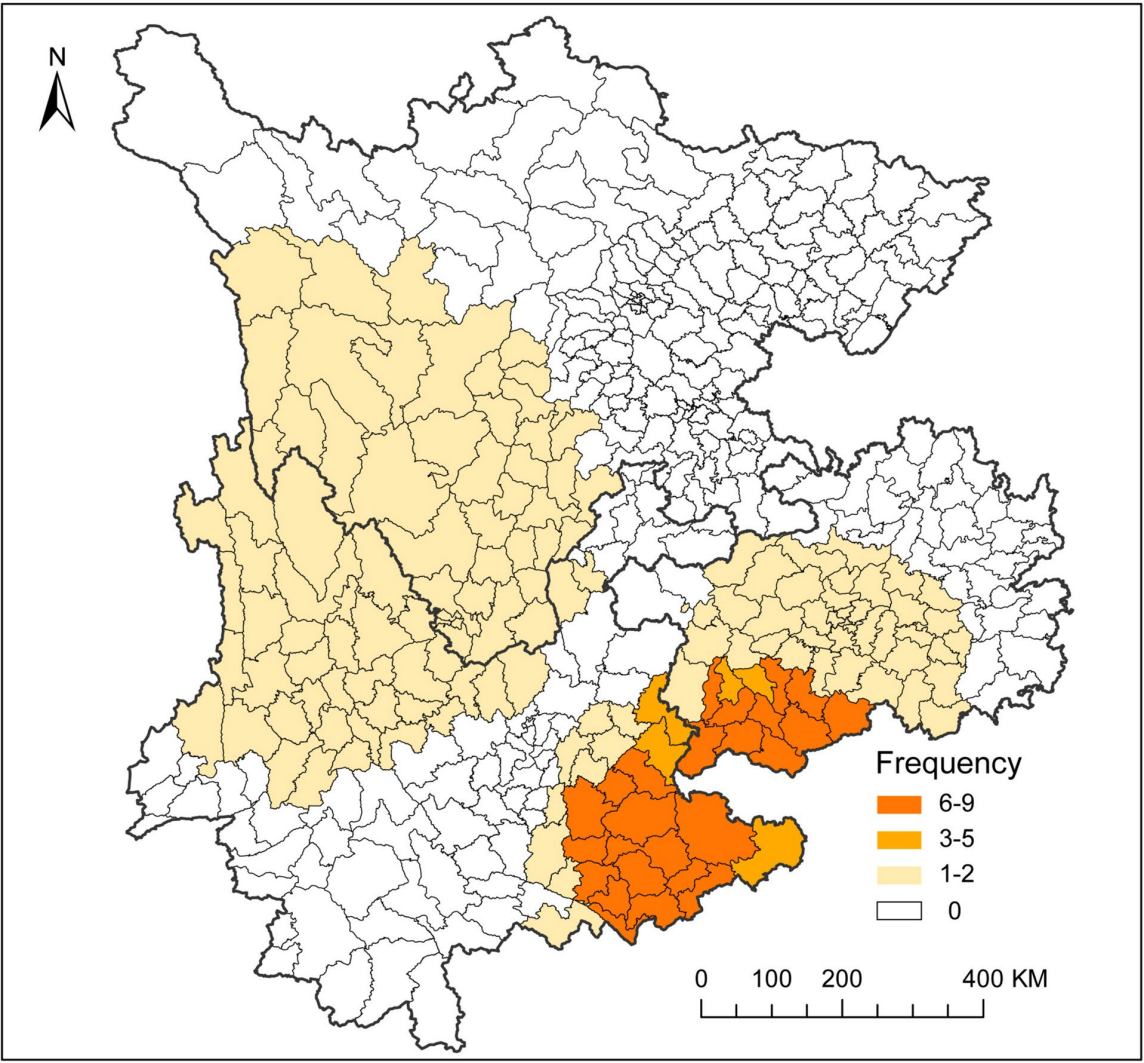
Frequency of most likely cluster occurrence from 2010 to 2020

The frequency of most likely spatial cluster occurrence was showed in Fig. 4. The southern region had the highest frequency (6–9) of leprosy clusters, which were composed of 14 counties in Yunnan Province, including Kaiyuan, Mengzi, Wenshan, Yanshan, Xichou, Mile, Guangnan, Malipo, Maguan, Qiubei, Shizong, Luxi, Hekou Yao Autonomous and Pingbian Miao Autonomous Counties; and 10 counties in Guizhou Province, consisting of Luodian, Xingyi, Xingren, Puan, Zhengfeng, Wangmo, Ceheng, Anlong, Zhengning Buyi and Ziyun Miao Autonomous Counties. The margins of high leprosy cluster counties and western counties had a low frequency of cluster occurrence. In contrast, the southeastern and northern regions had no most likely leprosy clusters during the study period.

### Space-time clusters of leprosy from 2010 to 2020

**Fig. 5:**
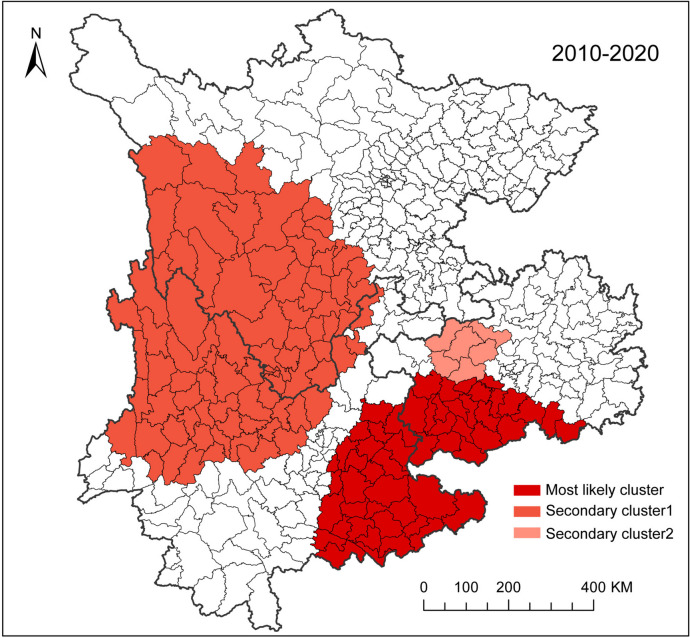
Spatial distribution of leprosy clusters detected by space-time scan statistics in southwest China from 2010 to 2020

In the space-time analysis, three spatiotemporal leprosy clusters were detected from 2010 to 2020 (Table 5; Fig. 5). Three clusters were statistically significant, including one most likely cluster and two secondary clusters. The most likely cluster was situated in the southeast area of the study region, which consisted of 52(13.00%) counties (29 counties in Yunnan, 23 counties in Guizhou), with RR = 4.46 (*P* < 0.001). The secondary cluster 1 was positioned in the western area, composed of 83 (20.75%) counties (RR = 3.79, *P* < 0.001), The secondary cluster 2 was located in the mid-east area, composed of 6 (1.50%) counties (RR = 6.26, *P* < 0.001).

**Table 5:** Leprosy clusters detected using the space-time scan statistics

Clusters	Time frame	Locations(N)	Observed	Expected	RR	LLR	*P*
Most likely cluster	2010/01/01-2013/05/31	52	707	177.77	4.46	472.40	< 0.001
Secondary cluster1	2010/01/01-2013/03/31	83	594	172.50	3.79	332.81	< 0.001
Secondary cluster2	2010/01/01-2012/07/31	6	191	31.58	6.26	187.02	< 0.001

Locations(N): Number of locations; *RR* Relative Risk, *LLR* Log likelihood ratio

## Discussion

Temporal and spatial statistic methods coupled with geographic information system were used to explore the spatiotemporal pattern of leprosy in southwest China from 2010 to 2020. Even though the NCDR had been declining over the past 11 years in the study area, spatial heterogeneity in the NCDR remains. Significant clusters were identified. The most likely cluster was predominantly centralized in the border areas between Yunnan and Guizhou Provinces, and the secondary clusters were always located in the western counties, the border areas between Yunnan and Sichuan Provinces.

In this study, both the number of newly detected leprosy cases and the NCDR showed a descending trend during the 11-year study period. The incidence or prevalence of leprosy also showed the same trend in other regions both domestic [14, 20] and abroad [21]. These achievements can be attributed to the vigorous promotion and wide use of MDT [5]. The establishment of relevant public health policies and investment of healthcare resources also play a crucial role [10] in eradicating leprosy burden. As the results show, the proportion of G2D in leprosy patients is declining but is still more than 10% annually, indicating delayed diagnosis in leprosy patients and continued transmission of *M. leprae* [22, 23]. Additionally, it is clearly noted that the proportion of G2D has fluctuated over time in Fig. 1a. This may be due to the publication of relevant policies, drawing more attention to leprosy, which could lead to an increase in the number of leprosy cases initially through active surveillance. Consequently, the number of leprosy cases and the number of patients with G2D increased in 2010–2012. However, there was a time lag between the promulgation of the policy and its entry into force [24]. In the case of this study, the start of the policy’s benefits may have been in 2013, and since then the number of leprosy cases and patients with G2D have continued to decline. This also reflects the effectiveness of the NLEP. More than 50% of the newly detected leprosy cases were determined as MB, which demonstrated the occurrence of disease precedes diagnosis, perpetuating the spread of disease [25]. A study based on Brazilian population-based cohort illustrated that the exposure of households to patients with leprosy may increase the risk of leprosy infection, especially in households with MB patients [26].

Global spatial autocorrelation analysis indicates that that the distribution of leprosy was most probably followed by a clustered pattern. The tendency of this pattern may primarily depend on the uneven distribution of factors that drive the transmission of leprosy [4, 27]. Previous studies indicated that the potential factors for leprosy infection are social factors, such as poverty [28], social vulnerability [21], and unequal access to healthcare resources [29]; and biological factors such as household contacts [30], undernutrition [31], helminth co-infections [32] and vitamin D deficiency [33].

Regardless of whether the purely spatial analysis or space-time analysis was used, the results revealed that the most likely leprosy clusters were mostly distributed in the border areas between Yunnan and Guizhou Provinces and the secondary clusters were always located in the western regions. Areas that persistently sustain a high leprosy burden need to be defined [34]. Detecting statistically significant leprosy clusters is a key step to the determine appropriate range of population for intervention [35]. Our results were consistent with previous studies, reflecting that leprosy was spatially clustered in certain geographic units [10, 21]. Hence, we need to focus on high-risk areas to prioritize control efforts, because these areas may be potential reservoirs of leprosy transmission [36]. Moreover, most likely and secondary clusters are always concentrated at the border areas between Yunnan and the other two provinces, which is why we should also focus on the migration of leprosy patients. From 2011 to 2018, 11.5% of newly detected leprosy cases in China were identified in populations migrating from areas where leprosy is traditionally endemic, from southwest China to relatively developed cities such as Beijing, Shanghai, and Guangzhou [37]. The purpose of their migration is to attain better development. During 2011 to 2019, 85.16% floating population cases in Zhejiang, China, came from southwest China [14]. These conditions suggest that the leprosy epidemic situation in the study area is still not promising. Thus, monitoring and tracing of cases in high endemic areas should be strengthened to facilitate the detection of leprosy cases. Furthermore, as shown by the result of space-time cluster, the time frame of the detected cluster is irregular, which revealed that there is no obvious seasonal trend in the transmission of leprosy. Therefore, the only way to eliminate leprosy as soon as possible is to strengthen surveillance and intervention in high-risk areas, not during high-risk time periods. Leprosy control policies can be adjusted and innovated to address these high burden areas [38]. However, the transmission route of *M. leprae* has not been completely understood [39], which is why further epidemiological models can be applied to more effectively identify areas with a high leprosy risk. An adequate exploration of the disease’s etiology and the local factors that increase the risk of leprosy is also required.

The study differs from other studies in that it used observational data over 11 years, thus providing evidence of the persistence of clusters in specific geographic aeras. The statistical methods used in this study were able to balance both Type I and II errors [40]. In the global spatial correlation analysis, the empirical Bayesian model was used to reduce random fluctuations resulting from rare events, particularly in counties with small populations or underreported cases [17]. As leprosy can be cured at the early stage, some measures could be taken in the areas characterized by clusters, such as enhancing the publicity and education to increase communities’ understanding of leprosy, strengthening active surveillance and contacts follow-up, especially in household contacts. Chemoprophylaxis is also an effective measure for this group such as single-dose rifapentine [41]. These are practical interventions that can promote early detection of cases. Most importantly, the findings of this study could provide evidence to guide leprosy control and prevention in southwest China, thus helping achieve the WHO Global Leprosy Strategy 2021–2030 targets [3]: a world with zero leprosy infection and disease, zero disability, and zero leprosy-related stigma and discrimination.

Although the main topic of our study is to explore the spatiotemporal pattern of leprosy in southwest China, the methods we used are robust and effective. Consequently, the same methods could be applied to determine clustering areas of leprosy in other regions to eliminate leprosy burden as soon as possible. These could also be applied to other infectious diseases to identify their high-burden areas to prioritize interventions.

Our study has some limitations. First, underreporting is a main challenge in disease surveillance [42]. The economy in our study area is less developed, which is why leprosy patients from rural and remote areas may be misdiagnosed and unreported. Therefore, the NCDR in this study may be underestimated. Second, this work is a retrospective study, and recall bias is difficult to avoid. Third, the outbreak of coronavirus disease (COVID-19) during our study period made the diagnosis of other infections difficult [43], so the leprosy data in 2020 may be affected.

## Conclusions

In this study, the spatiotemporal pattern of leprosy in southwest China was explored at the county level over an 11-year period. The temporal trends of leprosy continued to decline, but leprosy has a spatially heterogeneous distribution within the study area. The results illustrate that high-risk areas for leprosy are centralized in the border areas between Yunnan and two other provinces. According to the findings of this study, more attention needs to be paid to high-risk areas, because these may be potential reservoirs for the future infection of leprosy. High-risk areas should also be prioritized in the allocation of resources.

## Data Availability

All data analyzed in this study are from LEPMIS database in China. The datasets could be available from the corresponding author on a motivated request.

## Abbreviations

LEPMIS: Leprosy Management Information System
WHO: World Health Organization
MDT: Multidrug therapy
APC: Annual percent change
AAPC: Average annual percent change
G2D: Grade 2 Disability
MB: Multibacillary
CI: Confidence interval
RR: Relative Risk
COVID-19: Coronavirus disease 2019
